# Effects of dietary spirulina (*Arthrospores platensis*) on the growth performance and meat quality of broiler chickens

**DOI:** 10.1016/j.psj.2026.107282

**Published:** 2026-06-11

**Authors:** Emily Beckerman, Shreyash Patel, Darin C. Bennett, Siroj Pokharel, Mohammed Abo Ismail, Amy Laummert, Woo Kyun Kim, Iksoon Kang

**Affiliations:** a Animal Science Department, California Polytechnic State University, San Luis Obispo, CA, USA; b Food Science and Nutrition Department, California Polytechnic State University, San Luis Obispo, CA, USA; c Poultry Science Department, University of Georgia, Athens, GA, USA

**Keywords:** Spirulina, Broiler growth, Gut morphology, Gut microbiome, Meat quality

## Abstract

This study evaluated the effects of spirulina supplementation in broiler diets on growth performance of live birds and meat quality of carcasses. A total of 270 one-day-old Ross 708 broiler chicks were obtained from a local hatchery, weighed, and assigned to 18 floor pens (4′ × 4’ each, 15 birds/pen). Birds were fed corn and soybean meal (SBM) based diets supplemented with spirulina at inclusion levels of 0% (control), 2.5%, and 5% during the starter (0–2 weeks), grower (2–4 weeks), and finisher (4–6 weeks) phases. Body weight and feed intake were measured weekly to calculate body weight gain and feed conversion ratio (FCR). On 6th week, eighteen broilers (one bird per pen) were processed and evaluated for color, meat quality, and sensory attributes. No significant difference was observed among the dietary treatments for feed intake, FCR, or body weight throughout the study period. Spirulina supplementation had no significant effect on villi height (VH), crypt depth (CD), and VH/CD ratio (*P* > 0.05). However, spirulina supplementation positively influenced gut microbiome composition by promoting the abundance of fiber-fermenting and probiotic-associated bacteria while maintaining microbial balance. No significant effects were observed on chilling yield, pH, or shear force across the treatment groups (*P* > 0.05). A notable increase in yellowness (b*) was observed in carcass skin and skinless fillets (*P* < 0.05). Sensory analysis revealed that 2.5% spirulina enhanced appearance scores (*P* < 0.05), whereas 5% spirulina reduced juiciness (*p* < 0.05), with no change for flavor, tenderness, aftertaste, and overall. Based on these results, spirulina served as an alternative feed resource up to 5.0% inclusion with favorable effects on gut health and carcass yellowness.

## Introduction

Global demand for poultry meat continues to rise, driven by its reputation as a healthy, affordable, and palatable product (Connolly and Campbell, 2023; Papp et al., 2023; Sujiwo et al., 2018). With the increasing popularity of poultry and the projected global population of 9.7 billion by 2050, poultry meat production represented about 40% of global meat production in 2020 (FAO, 2023; United Nations, 2019).

This rapid growth presents sustainability challenges, particularly considering the reduction of arable land and water resources (Austic et al., 2013). Feed costs account for about 70% of poultry production expenses, promoting the poultry industry to seek alternative ingredients and improve feed conversion ratios (Cooke, 1987; El-Deek, 2020; Herath et al., 2023). Currently, broiler diets heavily rely on corn and soybean meals (SBM) as the main sources of energy and protein, resulting in competition with human food supply chains (Austic et al., 2013). This competition may exacerbate food security by increasing supply chain volatility, reducing stock levels, and contributing to price fluctuations (Masuda and Goldsmith, 2009).

Microalgae have emerged as promising alternative feed ingredients due to their rich nutritional profiles, including proteins, lipids, polysaccharides, vitamins, pigments, and bioactive enzymes (Dolganyuk et al., 2020; Moustafa et al., 2021; Nagappan et al., 2021). Among these, spirulina *(Arthrospira platensis)*, which contains 50–70% protein and a balanced amino acid profile comparable to soybean meal, has gained significant attention as a potential dietary supplement for poultry (Martins et al., 2021; Mullenix, et al., 2022; O’Lear Reid et al., 2024). Since the successful commercialization of spirulina in the late 70s, its production has expanded globally and currently represents more than 30% of the world’s microalgal biomass output (De la Jara et al., 2018).

Numerous studies have reported beneficial effects of spirulina supplementation in poultry diets, including improvements in growth performance, immune function, antioxidant capacity, and meat quality. For instance, Khan (2020) reported that spirulina supplementation at 0.2% significantly improved body weight and dressing yield in broilers compared to control. Similarly, Alghamdi et al. (2024) found that a combination of spirulina and Dunaliella at 0.2% enhanced growth performance, liver function, and lipid profiles in quail. These benefits are thought to be from enhanced probiotic colonization and improved nutrient absorption in the gastrointestinal tract (Camacho et al., 2019). However, no positive effect was observed on body weight gain, feed intake, and feed conversion ratio when spirulina supplement was used at 0.1% alone (El-Bahr et al., 2020).

A wide range of spirulina inclusion levels (0.1–21%) has been evaluated in broiler diets. However, most studies have focused on either low (< 1%) or high (≥ 15%) inclusion levels, whereas intermediate levels (1–14%) remained less studied, and in-depth research has not been conducted to cover live bird performance, gut microbiology, gut microbiome, carcass quality, and sensory evaluations. Although there are some conflicting results, high inclusion levels have occasionally led to suppressed performance (Costa et al., 2024; Evan et al., 2015; Pestana et al., 2020; Neumann et al., 2018; Ross and Dominy, 1990; Spinola et al., 2024; Venkataraman et al., 1994), whereas low inclusion levels often promote growth (Abdelfatah, 2024; Alwaleed, 2021; El Bahr et al., 2020; Herath et al., 2023; Moustafa et al., 2021; Park et al., 2018; Sharmin et al., 2020).

Given the limited number of studies evaluating intermediate inclusion levels (Abd El-Hady, 2022; Moujahed et al., 2011; Toyomizu et al., 2001 ), the objective of this study was to assess the effects of spirulina supplementation at 0% (no supplementation control), 2.5%, and 5% on broiler growth performance, gut morphology, microbiological parameters, carcass quality, and sensory attributes.

## Materials and methods

All experimental procedures were reviewed and approved by the California Polytechnic State University Institutional Review Board and Institutional Animal Care and Use Committee (Protocol #2502).

### Birds, feed preparation, and diets

**Fig. 1:**
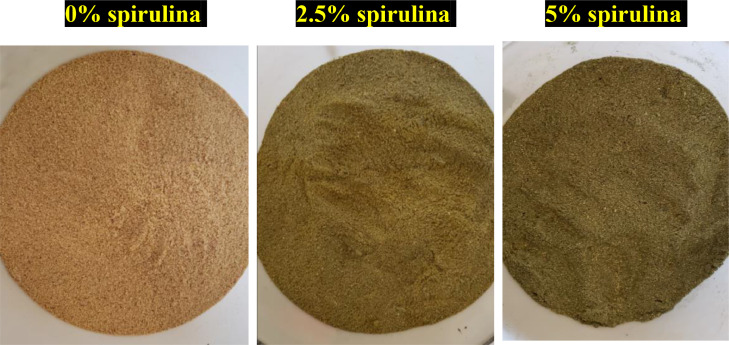
Corn-soybean meal-based diets with 0, 2.5, and 5% spirulina.

A total of 270 one-day-old Ross 708 broiler chicks were obtained from a commercial hatchery (Pitman Farms, CA) and reared at the California Polytechnic State University (Cal Poly) Poultry Center after being vaccinated against Newcastle, Marek’s disease, and Infectious Bronchitis at the hatchery. Upon arrival, they were sorted by body weight and placed to 18 floor pens (4′ × 4′ each, 15 birds/pen) for a similar average weight per pen. Pens were bedded with wood shavings, and both feed and water were provided *ad libitum*. Pens were randomly allocated to one of three dietary treatments (6 pens/treatment) consisting of corn-soybean meal-based mashed diets containing graded levels of spirulina at 0% (control), 2.5%, or 5%. The nutrient composition of the spirulina (Cyanotech Corporation, Kailua-Kona, HI, USA) used in this study is presented in Table 1. Apparent metabolizable energy and amino acid digestibility values were calculated using the availability coefficients determined for spirulina fed to broilers determined in O’Lear Reid et al. (2024). The diets were formulated and nutrients were analyzed for a three-phase feeding program (Table 2; Fig. 1 **in starter phase**): starter (d 1–14), grower (d 15–28), and finisher (d 29–42), all which met the nutrient guidelines for Ross 708 Broilers (Aviagen, 2019).

**Table 1:** Nutrient composition of the spirulina used in this studya.

Item	Concentration, as fed
Dry matter (%)	91.9
Gross energy (kcal/kg)	
Crude protein (%)	60.1
Crude fat (%)	4.4
Neutral detergent fiber (%)	3.3
Acid detergent fiber (%)	1.3
Ash (%)	9.0
Indispensable amino acids	
Arginine (%)	4.23
Histidine (%)	1.00
Isoleucine (%)	3.37
Leucine (%)	5.33
Lysine (%)	2.73
Methionine (%)	1.41
Phenylalanine (%)	2.74
Threonine (%)	3.01
Tryptophan (%)	
Valine (%)	3.47
Dispensable amino acids	
Alanine (%)	4.53
Aspartic acid (%)	5.63
Cysteine (%)	0.59
Glutamic acid (%)	7.73
Glycine (%)	2.98
Proline (%)	2.23
Serine (%)	2.87
Tyrosine (%)	2.80

a Cyanotech Corporation, Kailua-Kona, HI, USA.

**Table 2:** The coefficients of apparent ileal digestibility of amino acids of spirulina fed to broilers used to calculate digestible amino acid contenta.

Item	Concentration, as fed
Indispensable amino acids	
Arginine	0.904
Histidine	0.761
Isoleucine	0.768
Leucine	0.739
Lysine	0.945
Methionine	0.913
Phenylalanine	0.813
Threonine	0.714
Tryptophan	0.859
Valine	0.698
Dispensable amino acids	
Alanine	0.620
Aspartic	0.791
Cysteine	0.561
Glutamic	0.826
Glycine	0.667
Proline	0.805
Serine	0.781
Tyrosine	0.985

a (O’Lear Reid et al. 2024).

### Production performance

The birds were initially weighed before allocation while feed intake (**FI**, g/bird/d) and body weight (**BW**, g/bird) were measured once a week during the 6-week experimental period. Average daily gain (**ADG**, g/bird/d) and feed conversion ratio (**FCR**, g:g) were calculated, using the formula: ADG (g) = (final body weight – initial body weight)/number of days; FCR (g:g) = total feed intake/total body weight gain. The mortality of birds was recorded daily and used to correct the values of FCR.

### Broiler processing and tissue sampling

At the end of the 6 weeks, 18 broilers (one broiler/pen) were randomly selected, leg tagged, and subjected to feed withdrawal for 8 hours. These birds were then transferred from the Poultry Center to the Meat Processing Center at Cal Poly, where they were conventionally processed using electrical stunning for 3 s (40 mA, 60 Hz, 110 V), bleeding for 120 s, scalding for 120 s at 56.7 °C, and defeathering in a rotary drum picker for 120 s (Jeong et al., 2011). During evisceration, ileal tissue samples were collected from the midpoint of the ileum, rinsed with phosphate-buffered saline and immediately fixed in 10% neutral-buffered formalin for intestinal morphology analysis. Both ceca were collected aseptically, placed in sealed plastic bags, kept on ice, and stored at −20 °C until subsequent microbiome analysis.

Eviscerated carcasses were hung individually, wing tagged, and water chilled until their internal temperature reached 4.4 °C or below which was monitored by a digital thermometer/logger (ThermoData Thermocouple Logger KTC, ThermoWorks, American Fork, UT). For efficient and uniform chilling, the chilling water was agitated continuously using a submersible pump (4E-34NR, SupplyHouse.com). After chilling, carcasses were hung for 5 min and weighed to determine chilling yield: (postchill carcass weight/prechill carcass weight) x 100%. Breast fillets were deboned at 3 h post-mortem, and the right fillets were stored in sealed plastic bags on ice for cooking yield and shear force evaluations. The cranial portion of left fillets (1/4) were cut and immediately quickly frozen using liquid nitrogen for pH while the remaining portions (3/4) were frozen for sensory evaluation after labeling.

### Intestinal morphology

Fixed ileal tissues were processed and stained using the hematoxylin and eosin (H&E) staining (Feldman and Wolfe, 2014). Intestinal morphometric measurements were evaluated using the method of Teng et al. (2017). Villi height (VH) and crypt depth (CD) were measured using a light microscope at 5X (ileum) magnification (Leica DC500 camera, Leica Microsystems Inc., Buffalo Groove, IL). Measurements were taken from 5 randomly selected villi and crypts per slide, using the LAS v4.8 software (Leica Microsystems Inc.). The ratio of VH to CD was calculated from each sample.

### Cecal DNA extraction

DNA was extracted from cecal contents, using manufacturer protocols and the QIAamp PowerFecal Pro DNA Kits – Stool/Gut DNA Extraction kit (Qiagen, Germantown, MD) according to the manufacturer’s protocol. DNA concentrations were measured using a NanoDrop™ One/OneC Microvolume UV–vis Spectrophotometer (ThermoFisher Scientific, Pittsburgh, PA). Extracted DNA samples were sent to LC Sciences (Houston, TX, USA) for microbial sequencing.

### Microbial sequencing, identification, classification, and quantification analysis

Microbial sequencing was conducted using the 16S rDNA sequencing method as described by Abo-Ismail et al. (2024). Briefly, primers were tagged at their 5′ ends with unique barcodes for each sample, followed by sequencing with universal primers. Polymerase chain reaction (PCR) amplification was carried out in a 25 μL reaction mixture containing 25 ng of template DNA under the following conditions: an initial denaturation step at 98 °C for 30 s, followed by 32 cycles of denaturation at 98 °C for 10 s, annealing at 54 °C for 30 s, and extension at 72 °C for 45 s. A final extension was performed at 72 °C for 10 min. The PCR products were verified using 2% agarose gel electrophoresis, purified with AMPure XT beads (Beckman Coulter Genomics, Danvers, MA, USA), and quantified using Qubit (Invitrogen, USA).

For sequencing, amplicon pools were prepared using primers (341F: CCTACGGGNGGCWGCAG, 805R: GACTACHVGGGTATCTAATCC) designed to target the V3 and V4 regions of the 16S rDNA, generating amplicons approximately 465 bp in length. These variable regions of 16S rRNA were amplified to create the sequencing library. Following the manufacturer's guidelines, the amplified library underwent sequencing on the NovaSeq platform with paired-end reads (2 × 250 bp). The raw data was processed and filtered under specific conditions to obtain high-quality, clean data. Merging of paired-end reads, quality control, and chimera removal were performed using DADA2 (Divisive Amplicon Denoising Algorithm), Version 1.20 (Callahan et al., 2016).

The cleaned data was then used to generate operational taxonomic units (OTUs), construct the final feature table and feature sequences, and conduct diversity analysis, species classification, and differential analysis. Cluster analysis was performed using CD-HIT, with a sequence similarity threshold of 97% within each cluster. The longest tag within each cluster was designated as the representative sequence.

#### Cooking yield and shear force

To measure cooking yield and shear force, fillets were placed on a stainless-steel rack, covered with aluminum foil and cooked to an internal temperature of 76 °C in a convection oven (36S-Y1A Wolf Challenger XL Range, ITW Food Equipment Group LLC, Glenview, IL), following the USDA-Food Safety and Inspection Services (2001) guidelines. Cooking yield was calculated by the following equation: (cooked breast weight)/(raw breast weight) x 100. Shear force was evaluated to assess meat tenderness by the razor-blade method described by Cavitt et al. (2004), using a texture analyzer (TAHDi, Texture Technologies Corp., Scarsdale, NY) that was calibrated with a 25-kg load cell. The razor blade (height: 24 mm, width: 8 mm) was set to penetrate the samples at 10 mm/s, triggered by a 10-g contact force. The maximum shear force (N) required to penetrate each sample was recorded, and the average of the two measurements was calculated to represent the fillet’s tenderness.

#### pH

The pH of the fillets was measured using a pH electrode (Model 13620631, Fisher Scientific Inc., Houston, TX) connected to a pH meter (Accumet AR15, Fisher Scientific Inc., Pittsburgh, PA), following the iodoacetate method described by Sams and Janky (1986). For each sample, 2.5 g of breast fillet tissue was pulverized and homogenized with 25 mL of a 5-mM sodium iodoacetate solution containing 150 mM potassium chloride for 30 seconds. The pH meter was calibrated at pH 4.0 and 7.0 before measurement.

#### Color

Commission Internationale de l'Éclairage (CIE) values (lightness L*, redness a*, and yellowness b*) were measured using a chromameter (CR-400, Konika Minolta Sensing Inc., Osaka, Japan) with an 8-mm aperture and illuminant C after calibration, using a white calibration plate (L*, 97.28; a*, −0.23; b*, 2.43). For carcass skin color, four readings were taken on the skin surface (2 on right and 2 on left), using the areas of no visible blood, bruising, physical defects, or filled blood vessels (Fletcher, 2002). The skin was removed from the carcass to measure skinless fillet color in a similar way as explained above.

#### Consumer sensory

A total of 92 consumer panelists were recruited from students, staff, and faculty members at Cal Poly, screened for dietary restrictions and requested to consent to participate in the evaluation. Frozen fillets were thawed overnight in a refrigerated room at 4.4 °C and cooked as described before. After cooking, the central portion of each filet was cut to approximately 5 × 6 cm, wrapped with aluminum foil, and kept at 60 °C in a warmer (RO230-C, 22-Quart Roaster Oven, Rival, Milford, MA) until the sensory evaluation was completed within 2 h. Upon serving, the prepared breast was cut again into 4 pieces (approximately 12–15 g). One piece from each treatment was labeled with a 3-digit random number and placed on a polyfoam tray with a cover. The tray, with 3 samples, was randomly presented to each panelist. Both filtered water and unsalted crackers were provided for mouth cleansing between samples. Sensory evaluations were conducted in individual booths equipped with a touchscreen computer and controlled lighting. Questionnaires were prepared and data were collected using RedJade Sensory Software (RedJade Software Solutions, Mountain View, CA, USA). Panelists were asked to evaluate the samples for flavor, texture, juiciness, and overall acceptability on a 9-point hedonic scale (9 = like extremely; 1 = dislike extremely). They were also encouraged to make comments on their decisions.

### Statistical analysis

Statistical analyses were conducted using SAS 9.4 software (SAS Institute Inc., Cary, NC). Production performance (body weight, ADG, FI, and FCR) were analyzed with a one-way repeated measured ANOVA, with dietary treatment as the main effect and age (week) as the repeat, and pen as the experimental unit. Intestinal morphology data were analyzed with one-way ANOVA, with dietary treatment as the main effects and pen as the experimental unit. For sensory analysis, a mixed model analysis of variance was used for separation of means with Tukey’s test at *P* < 0.05. Data were reported as means ± SEM, and statistical significance was accepted at *P* < 0.05.

## Results and discussion

### Production performance

**Fig. 2:**
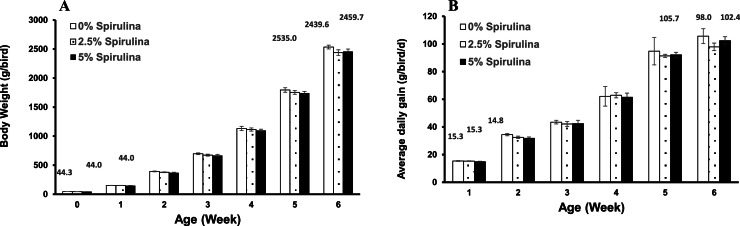
Effect of dietary *spirulina* sp. on body weight (A) and daily gain (B) of Ross 708 broiler chickens over 6 weeks, (*n* = 18 pens/dietary treatment). Data are presented as means ± SEM.

The initial body weight of the one-day-old chicks was 44.1 ± 0.2 g/chick, which is consistent with previous studies (Alwaleed et al., 2021; Abdelfatah et al., 2024; Spinola et al., 2024). Final body weight at six weeks ranged from 2440 to 2535 g/bird, with no significant differences for body weight or average daily gain at any week, regardless of treatment (*P* > 0.05) (Fig. 2**A, B**). Moujahed et al. (2011) reported no significant difference in body weight and daily gain, using a similar range of spirulina supplementation.

In general, broiler body weight increases proportionally as spirulina supplementation was raised up to 1%, with a significant improvement at inclusion levels of ≥ 0.5% (Alwaleed et al., 2021; Abdelfatah et al., 2024). The improvement is expected due to the rich nutrient profile, including amino acids, phycocyanins, tocopherols, β-carotenes, and chlorophyll (Abd El-Baky et al., 2003). According to Khan et al. (2020), the increase of feed intake and weight gain with 0.2% spirulina were attributed to its taste, odor, and ease of digestion. Ross and Dominy (1990) reported that birds who were fed 1.5, 3.0, or 6.0% had significantly higher body weights than those who were fed 12% spirulina (*P* < 0.05), with the control group showing an intermediate value. At higher spirulina inclusions (10–15%), studies reported significant weight losses and increased incidence of foot dermatitis (Mullenix et al., 2022; Costa et al., 2024; Spinola et al., 2024). Pestana et al. (2020) suggested that 15% spirulina inclusion impairs performance due to increased digesta viscosity, likely caused by gelation of indigestible microalgal proteins or poorly digestible spirulina proteins as suggested by Evnas et al. (2015).

**Fig. 3:**
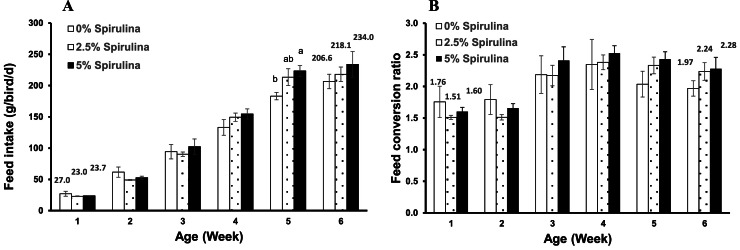
Effect of dietary *spirulina* sp. on feed intake (A) and feed conversion ratio (B) of Ross 708 broiler chickens over 6 weeks (*n* = 18 pens/dietary treatment). Data are presented as means ± SEM.

Broiler body weight generally increased with spirulina supplementation up to 3.0%, plateaued at 4.0%, and declined at 12% when spirulina was supplemented at levels ranging from 1% to 12%. Although there was no significant difference statistically between control and spirulina-fed birds at 2.5% and 5% (Fig. 2) (*P* > 0.05), numerical values indicated the trend of less bird performance with increased spirulina supplementation by decreasing body weight and daily weight gain while increasing feed intake and feed conversion ratio (Fig. 2, Fig. 3). These results are presumed due to the disadvantages of digesta viscosity outperformed the nutritional advantages in live birds.

#### Feed intake (g) and feed conversion ratio (feed/grain)

In accordance with the body weight gain, there is no significant difference for feed intake, regardless of treatment, except the 5th week showing a significantly higher feed intake in spirulina supplementation than control (*P* < 0.05) (Fig. 3**A**).

A similar trend of no significant difference was observed for feed conversion ratio (Fig. 3**B**). Unlike our study, however, Moujahed et al. (2011) reported reduction in FCR using spirulina diets at 1, 2.5, and 5% over the control diet. In a study evaluating diets containing 0, 6, 11, 16, and 21% spirulina*,* no significant differences in live weight gain or feed intake were observed, except the 21% level, where both parameters were significantly reduced (*P* < 0.05) (Evans et al., 2015). Khan et al. (2020) found that increasing spirulina levels from 0 to 0.2% led to proportional increases in feed intake and lower FCR values over controls. However, Abdelfatah et al. (2024) reported no significant differences in feed intake at similar spirulina levels (0, 0.1, 0.3, and 0.5%) (*P* > 0.05) although FCR was significantly lower at 0.3 and 0.5% incorporations (*P* < 0.05).

Using higher spirulina level (15%), previous studies indicated that spirulina was associated with feed intake reduction and FCR increase, indicating poorer feed efficiency (Costa et al., 2024; Pestana et al., 2020). spirulina is a blue-green algae, taxonomically classified as cyanobacteria (or Cyanophyceae), with multilayer cells including peptidoglycan and lipopolysaccharide (Cohen, 1997; Drews, 1973). These rigid structures are indigestible by monogastric animals, potentially impairing intestinal function and overall growth performance. This presents a challenge for incorporating microalgal nutrients into poultry diets especially at high supplementations (Austic et al., 2013; Coelho et al., 2019; Lum et al., 2013; Madeira et al., 2017).

#### Gut morphology

Broiler weight gain and feed conversion ratio are closely associated with intestinal health and nutrient absorption. Broiler weight gain and feed conversion ratio are closely linked to intestinal health and nutrient absorption. Previous studies have shown that increased villus height and reduced crypt depth enhance nutrient uptake and improve digestive efficiency within the gastrointestinal tract (Bafundo et al., 2021; Wang et al., 2025). The effects of spirulina on intestinal morphology were evaluated by measuring villus height (VH), crypt depth (CD), and the VH/CD ratio. No significant difference was found in VH, CD, and the VH/CD ratio regardless of spirulina treatment (*P* > 0.05) (Table 3). Similar results of no difference in VH, CD, and VH/CD were observed with spirulina supplementation at 0, 3, and 6% levels (Abd El-Hady et al., 2022).

**Table 3:** Ingredient composition and calculated nutrient analysis of the Starter (1–14 d), Grower (15–28 d), and Finisher (29–42 d) diets containing graded levels of spirulina (0%, 2.5%, 5%). Ingredient composition and calculated analysis are reported on as a fed basis.

	Dietary treatment								
	Starter diets (1–14 d)^a^			Grower diets (15–28 d)			Finisher diets (29–42 d)		
	0%	2.5%	5%	0%	2.5%	5%	0%	2.5%	5%
Ingredients (%)									
Corn	48.56	48.97	49.37	49.35	49.75	50.13	46.99	47.59	48.16
Soybean meal	39.25	36.38	33.50	32.83	29.95	27.10	33.09	29.47	25.71
Spirulina	0.00	2.50	0.50	0.00	2.50	5.00	0.00	2.50	5.00
Corn DDGS	5.00	5.00	5.00	10.00	10.00	10.00	10.00	10.00	10.00
Wheat midds	0.00	0.00	0.00	0.00	0.00	0.00	2.26	2.79	3.46
Vegetable fat	2.50	2.39	2.28	3.59	3.48	3.38	3.99	3.89	3.79
Limestone	1.32	1.35	1.37	1.27	1.30	1.33	1.17	1.21	1.25
Mono-Dicalcium Phosphate blend	1.55	1.58	1.61	1.27	1.30	1.32	1.10	1.13	1.16
Salt	0.32	0.32	0.32	0.30	0.30	0.30	0.29	0.30	0.30
DL-Methionine	0.39	0.38	0.37	0.33	0.32	0.31	0.27	0.26	0.26
L-Lysine HCl	0.11	0.14	0.17	0.09	0.12	0.16	0.00	0.01	0.08
L-Theonine	0.13	0.13	0.12	0.10	0.09	0.09	0.03	0.03	0.04
Vitamin premix^b^	0.50	0.50	0.50	0.50	0.50	0.50	0.50	0.50	0.50
Trace mineral premix^c^	0.30	0.30	0.30	0.30	0.30	0.30	0.30	0.30	0.30
Nicarb 25%	0.08	0.08	0.08	0.08	0.08	0.08	0.00	0.00	0.00
									
Calculated analysis									
AME (kcal/kg)	3000	3000	3000	3100	3100	3100	3100	3100	3100
Dry matter (%)	88.06	88.12	88.17	87.97	88.03	88.08	88.02	88.07	88.12
Crude protein (%)	23.47	23.71	23.95	21.86	22.11	22.36	22.00	22.00	22.00
Crude Fat (%)	5.30	5.27	5.24	6.59	6.56	6.54	7.00	7.00	7.00
NDF (%)	8.99	8.81	8.62	10.10	9.91	9.73	10.76	10.73	10.75
Ca (%)	0.96	0.96	0.96	0.87	0.87	0.87	0.81	0.81	0.81
Available P (%)	0.48	0.48	0.48	0.43	0.43	0.43	0.40	0.40	0.41
Digestible Methionine (%)	0.70	0.70	0.70	0.63	0.63	0.63	0.57	0.57	0.58
Digestible TSAA (%)	0.99	0.99	0.98	0.90	0.89	0.89	0.85	0.84	0.84
Digestible Lysine (%)	1.30	1.30	1.29	1.16	1.15	1.15	1.10	1.06	1.06
Digestible Threonine (%)	0.86	0.86	0.86	0.90	0.77	0.77	0.71	0.71	0.71

a The 2.5% starter diet was prepared by blending 50:50 the 0% and 5% starter diets.

b Supplied per kg diet: vitamin A, 20,000 IU; vitamin D_3_, 5000 IU; vitamin E, 20 IU; vitamin K_3_, 6 mg; thiamine, 2.0 mg; riboflavin, 8.0 mg; niacin, 60 mg; pantothenic acid, 16.0 mg; vitamin B_6_, 4 mg; biotin, 50 μg; folic acid, 1.0 mg; vitamin B_12_, 30 μg; Choline chloride, 800 mg.

c Supplied per kg diet: manganese (MnSO_4_), 120 mg; zinc (ZnSO_4_), 72 mg; iron (FeSO_4_), 30 mg; copper (CuSO_4_), 6 mg; selenium (Na_2_SeO_3_), 0.24 mg.

Using lower-level spirulina (0.1–0.2%), however, Khan et al. (2020) and Mahmoud et al. (2025) reported that broilers exhibited well-developed intestinal villi, suggesting enhanced intestinal absorption and improved gut morphology at optimal doses. These positive effects on gut morphology are expected from the function of spirulina’s a natural antioxidant, anti-inflammation, and gut regulating properties (Deng and Chow, 2010; Khambaulai et al., 2009; Rasool et al., 2006; Sugiharto et al., 2018). Excessive production of reactive oxygen species and free radicals can damage organs and contribute to tumor formation, and neurodegenerative diseases (Lopez-Otín et al., 2013; Tezil and Basaga, 2014). Spirulina’s antioxidant capabilities may help mitigate organ damage via neutralizing free radicals and proving radioprotective effects under oxidative stressful conditions (Khambaulai et al., 2009; Khan et al., 2020; Han et al., 2021). Increased villus height accompanied by reduced crypt depth has also been reported in broilers fed mannan-oligosaccharides (MOS), a type of prebiotic, suggesting similar gut health benefits (Chee et al., 2010; Teng et al., 2021).

Feeding 15% spirulina, however, Pestana et al. (2020) and Spinola et al. (2024) showed increased viscosity in ileum contents and longer intestinal segments (duodenum, jejunum, ileum, and caecum) compared to control birds. The higher digesta viscosity, combined with lower digestibility, likely reduced the nutritional value of spirulina proteins due to their resistance to their endogenous peptidases in broilers (Pestana et al., 2020). The values of thiobarbituric acid reactive substances (TBARS) have been used as markers of oxidative stress, with lower values indicating greater oxidative stability.

#### Gut microbiome

Elimination of potential pathogens from intestinal tract in animals enhances their physiological functions such as digestion, absorption, and growth (Jeong and Kim, 2014; Savage and Zakrzewska, 1996). Spirulina supplementation was shown to modulate gut microbiota, influencing host health, metabolic efficiency, and immune function (Flint et al., 2012; Geller et al., 2017; Neyrinck et al., 2017; Yusuf et al., 2016). Shanmugapriya et al. (2015) reported that 1% spirulina supplementation increased the population of lactic acid bacteria and yeast in the cecum while decreasing *E. coli* populations. Methanolic and aqueous extracts of spirulina were also found to stimulate the growth of *Lactococcus lactis* and inhibit harmful microorganisms such as *Candida albicans, Staphylococcus aureus, E. coli, Pseudomonas aeruginosa, Salmonella Typhi*, and *Klebsiella pneumonia* (De Mule, et al., 1996; Kaushik and Chauhan, 2008).

**Fig. 4:**
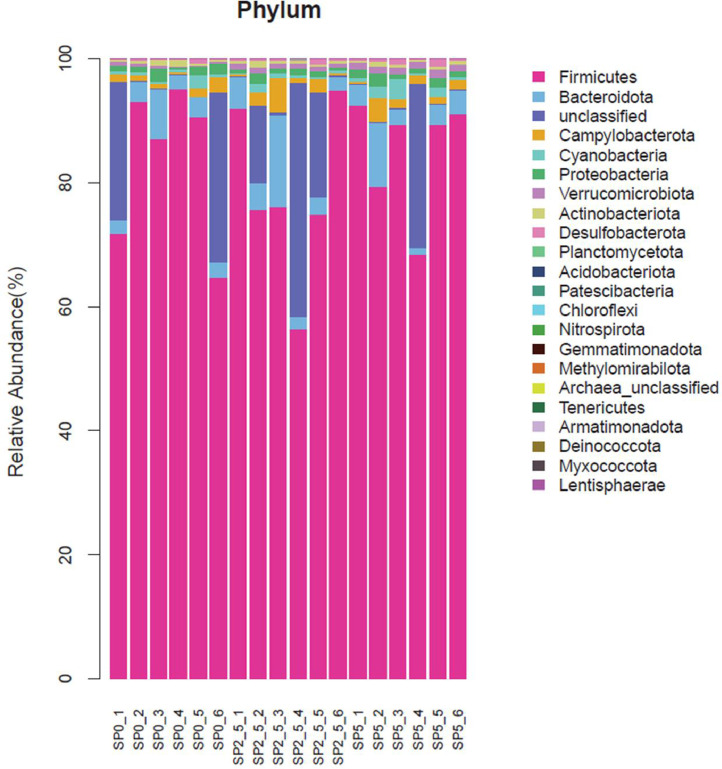
Phylum-level relative abundance of gut microbiota in experimental groups.

**Fig. 5:**
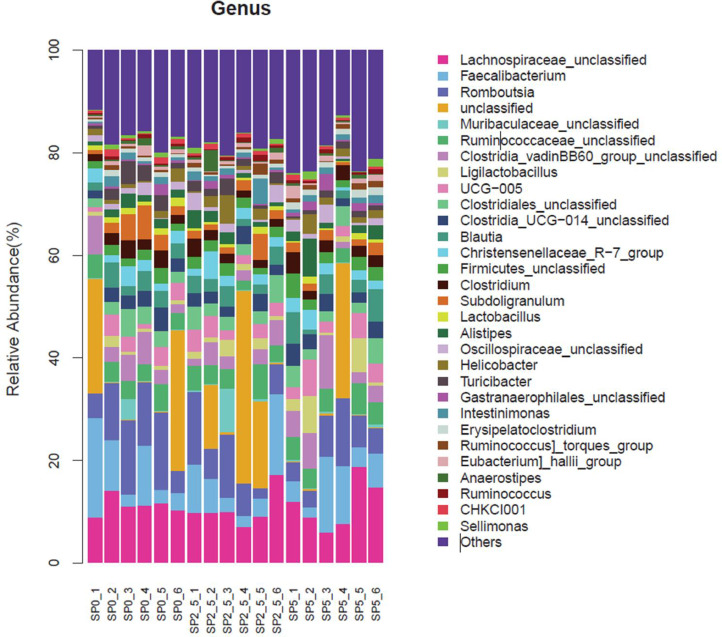
Genus-level relative abundance of gut microbiota in experimental groups.

In our study, phylum and genus relative abundance distribution of spirulina supplementation and other diet treatments are illustrated in Fig. 4, Fig. 5. At the phylum level, most abundant groups were Firmicutes, Bacteroidetes, Verrucomicrobia, and Proteobacteria, with a notable dominance by anaerobic bacteria (0.75 relative abundance) over aerobic bacteria (0.067 relative abundance) (Fig. 4). Higher spirulina concentrations led to an increase in the relative abundance of Firmicutes and Bacteroidetes, suggesting a positive impact on gut microbial balance (Fig. 4). Similar findings were observed in mice, where spirulina fed at a low dose (1.5 g/kg) increased the ratio of Firmicutes and Bacteroidetes while those fed a high dose (3.0 g/kg) showed a diminished effect (Hu et al., 2019).

At genus levels, bacterial microbes such as Lactobacillus, Ruminococcus, and Blautia were detected, all of which play roles in fiber digestion and gut health. Principal component analysis revealed distinct clustering patterns between control and spirulina-fed groups, with the most pronounced microbiome shift at higher spirulina concentration (Fig. 5). These findings suggest that spirulina supplementation at 5% may promote a beneficial gut microbiome composition by promoting fiber-fermenting and probiotic-associated bacteria while maintaining microbial population balance. Similar results were reported by Bhowmik et al. (2009), who documented spirulina’s probiotic effects and its antimicrobial activity against human pathogenic bacteria.

#### Chilling yield, pH, shear force

There were no significant differences in chilling yield, fillet pH, or shear force, regardless of spirulina inclusion level (*P* > 0.05) (Table 4). The chilling yield (102–107%) in this study is consistent with previous findings following water immersion chilling (Jeong et al., 2011; Kawamura et al., 2023; Metheny et al., 2019). Although whole carcass weight was not evaluated, Toyomizu et al. (2001) reported no significant difference in breast fillet weight after spirulina was supplemented at 0, 4, and 8%. The value (6.2) of fillet pH at 3 h postmortem (pH_3h_) in this study is comparable to the reported value (6.14) for fillets subjected to water immersion chilling (Sansawat et al., 2014). Moujahed et al. (2011) observed a linear decline in average muscle pH from 6.0 at 15 min to 5.67 at 3 h, and 5.57 at 24 h postmortem, with no significant difference among spirulina inclusion levels ranging from 0 to 5%. In contrast, Altmann et al. (2018) reported that diets containing approximately 10% spirulina resulted in higher pH at 24 h, along with greater cooking yield and similar shear force compared with control fillets. However, at 15% inclusion level, Costa et al. (2024) observed a significant reduction in breast muscle pH from 5.66 in control birds to 5.48 in spirulina birds.

**Table 4:** Effects of dietary spirulina on intestinal morphologya.

Parameter	Spirulina			
	0%	2.5%	5%	SE
**Villus height (VH, µm)**	1209.2	1141.0	1196.1	±52.0
**Crypt depth (CD, µm)**	284.7	269.1	279.8	±18.4
**VH/CD ratio**	4.35	4.31	4.38	±0.32

a Number of oberservations in each parameter, *n* = 6.

In this present study, mechanical shear force (12.7–13.0 N) did not differ among treatments, consistent with the findings of Sansawat et al. (2014), who evaluated breast fillets at 3 h postmortem following water immersion chill. Meat tenderness is generally influenced by several factors such as proteolytic enzyme, the degree of rigor mortis (or sarcomere length), and connective tissue content (Cross et al., 1980; Koohmaraie and Geesink, 2006). Spirulina supplementation at 2.5–5.0% in the diet did not affect breast fillet tenderness in this study.

#### Color analysis

The color and visual appearance of broiler carcasses are important quality attributes because they primarily influence consumers’ first impressions and purchasing decisions (Fletcher, 2002; Jeong et al., 2011). Spirulina contains distinctive natural pigments, including carotenoids, chlorophylls, and phycocyanins, which impart orange, green, and blue colors, respectively (Chamorro-Cevallos, 2016). The color characteristics of both skin-on and skin-off breast fillets were evaluated. No significant differences were observed in L* and a* values among treatments, whereas b* value increased with increasing dietary spirulina inclusion (Table 5). These results are in line with previous reports showing no difference in L* and a* values but a significant increase in b* value in broilers supplemented with 2.5, and 5% spirulina (Moujahed et al., 2011).

**Table 5:** Effect of spirulina supplementation on meat quality parameters in broiler breast filletsa.

Parameter	Spirulina			*P*- value
	0%	2.5%	5%	
**Chilling Yield (%)**	102.1 ± 1.10	106.9 ± 1.10	102.8 ± 1.10	0.4096
**pH_3h_**^b^	6.160 ± 0.14	6.162 ± 0.14	6.181 ± 0.14	0.9410
**Shear Force (N)**	12.743 ± 0.47	12.842 ± 0.74	13.031 ± 0.74	0.7985

a Values expressed as standard error of the means ± SEM (*n* = 18).

b Values measured at 3 h postmortem.

The linear increase of b* value is due to the accumulation of carotenoid pigments, such as β-carotene and zeaxanthin, derived from spirulina (Fernandes et al., 2020; Pestana et al., 2020; Moujahed et al., 2011; Mullenix et al., 2022; Spinola et al., 2024; Toyomizu et al., 2001). Although no significant differences were detected in a* value among treatments, the numerical trend toward increased redness may be associated with greater myoglobin or iron deposition in carcasses from birds fed mineral-rich spirulina diets (Toyomizu et al., 2001). Similarly, although L* value did not differ significantly, the numerically lower L value observed in birds fed the 5% spirulina diet over the control may reflect increased pigment deposition, resulting in darker breast meat (Toyomizu et al., 2001).

#### Sensory attributes

Table 6 shows the results of consumer sensory attributes evaluated by 92 sensory panels. No significant differences were observed for color, aroma, flavor, texture, tenderness, aftertaste, or overall (*P* > 0.05). Although increased yellowness (b*) was detected in raw skin and breast fillets (Table 5), this difference was not perceived by the consumer panel, potentially because yellowness was not retained after cooking. Unlike red meat, a bright red color is not desirable while a bluish-white color is considered normal and acceptable for skinless cooked broiler breast meat (Nam et al., 2016) (Table 7).

**Table 6:** Effect of spirulina supplementation on meat quality parameters in broiler breast filletsd.

Parameter	Spirulina				*P*-value
		0%	2.5%	5%	
L* (Lightness)	**Skin-on**	74.31 ± 1.6^a^	74.52 ± 1.6^a^	73.35 ± 1.6^a^	0.0746
	**Skin-off**	66.07 ± 2.2^a^	65.67 ± 2.2^a^	65.20 ± 2.2^a^	0.4936
a* (Redness)	**Skin-on**	2.59 ± 1.3^a^	2.81 ± 1.3^a^	3.19 ± 1.3^a^	0.4058
	**Skin-off**	−0.11 ± 1.0^a^	0.03 ± 1.0^a^	0.47 ± 1.0^a^	0.2199
b* (Yellowness)	**Skin-on**	3.97 ± 2.1^a^	7.29 ± 2.1^b^	9.58 ± 2.1^c^	< 0.0001
	**Skin-off**	1.42 ± 1.1^a^	4.34 ± 1.1^b^	7.24 ± 1.1^c^	< 0.0001

a–c Within a row, means with different letters are significantly different (*P* < 0.05).

d Values expressed as standard error of the means ± SEM (*n* = 18).

**Table 7:** Effect of spirulina supplementation on meat quality parameters in broiler breast filletsc.

Parameter	Spirulina			*P*-value
	0%	2.5%	5%	
**Appearance**	5.63 ± 1.9ᵇ	6.25 ± 1.9^a^	5.63 ± 2.0ᵇ	0.046
**Color**	5.68 ± 1.9	6.08 ± 1.9	5.75 ± 1.9	0.315
**Aroma**	6.00 ± 1.5	6.07 ± 1.4	6.03 ± 1.5	0.417
**Flavor**	6.35 ± 1.5	6.41 ± 1.7	6.10 ± 1.7	0.498
**Texture**	5.27 ± 2.1	5.44 ± 2.2	5.46 ± 2.2	0.542
**Tenderness**	5.08 ± 2.3	5.22 ± 2.2	5.09 ± 2.3	0.712
**Juiciness**	5.31 ± 1.8^a^	5.40 ± 1.9^a^	4.73 ± 2.0ᵇ	0.027
**Aftertaste**	5.67 ± 1.5	5.74 ± 1.5	5.45 ± 1.5	0.408
**Overall**	5.71 ± 1.9	5.98 ± 2.0	5.94 ± 2.0	0.356

a–b Within a raw, means with different letters are significantly different (*P* < 0.05).

c Values expressed as standard error of the means ± SEM (*n* = 92).

A more appealing score was observed in fillets from birds fed 2.5% spirulina, whereas a lower juiciness score was observed with 5% spirulina over the control (*P* < 0.05%). In a study evaluating spirulina supplementation levels ranging from 0 to 5%, Moujahed et al. (2011) similarly reported no significant difference in sensory attributes, regardless of treatment. However, Venkateswarlu et al. (2023) reported that dietary supplementation with 1 and 2% spirulina improved meat tenderness, juiciness, and overall acceptability compared with the control, whereas higher inclusion levels (15%) did not result in sensory differences relative to the control (Pestana et al., 2020).

## Conclusion

Microalgae, such as spirulina, show considerable promise as alternative feed ingredients in the poultry industry because of their nutritional value, growth promoting properties, and potential health benefits. In this study, dietary supplementation with spirulina at 2.5 and 5% did not result in significant differences in feed intake, FCR, or overall growth performance compared to the control group. However, spirulina did positively influence gut microbiome composition without altering the total bacteria population. Previous studies have shown that spirulina supplementation at levels below 1% can enhance broiler performance, whereas inclusion levels between 5 and 10% generally have no measurable effect, and levels at or above 12–15% may negatively impact growth performance and bird health (Khan et al., 2020; Pastena et al., 2020; Toyomizu et al., 2001 ). Given relatively high costs of spirulina, it is essential to evaluate its use in broiler diets not only for biological benefits – such as health enhancement, improved growth, reduced mortality, and better carcasses quality – but also for cost-effectiveness to ensure economic viability. Based on the lack of significant improvements in broiler performance in this study, spirulina supplementation levels at 2.5% and lower may be more appropriate for practical application.

## CRediT authorship contribution statement

**Emily Beckerman:** Writing – original draft, Investigation, Formal analysis, Data curation. **Shreyash Patel:** Writing – original draft, Investigation, Formal analysis, Data curation. **Darin C. Bennett:** Writing – review & editing, Supervision, Data curation, Conceptualization. **Siroj Pokharel:** Writing – review & editing, Methodology, Formal analysis. **Mohammed Abo Ismail:** Writing – original draft, Investigation, Formal analysis, Data curation. **Amy Laummert:** Supervision, Methodology, Data curation. **Woo Kyun Kim:** Methodology, Investigation, Formal analysis. **Iksoon Kang:** Writing – review & editing, Supervision, Project administration, Investigation, Funding acquisition, Data curation, Conceptualization.

## Disclosures

There is no potential conflict of interest for the authors in the work.

## References

[bib0001] Abd El-Baky H.H., El-Baz F.K., El-Baroty G.S. (2003). Spirulina species as a source of carotenoids and α-tocopherol and its anticarcinoma factors. Biotechnology.

[bib0002] Abd El-Hady A.M., Elghalid O.A., Elnaggar A.S., Abd El- Khalek E. (2022). Growth performance and physiological status evaluation of Spirulina platensis algae supplementation in broiler chicken diet. Livest. Sci..

[bib0003] Abdelfatah S.H., Yassin A.M., Khattab M.S., Abdel-Razek A.S., Saad A.H. (2024). Spirulina platensis as a growth booster for broiler; insights into their nutritional, molecular, immunohistopathological, and microbiota modulating effects. BMC Vet. Res..

[bib0004] Abo-Ismail M., Sadek A.A., Humagain M., Banjara K., N, Pokharel S. (2024). Spatiotemporal distribution of environmental microbiota around animal farms adjacent to produce fields in central coast California. Food Microbiol..

[bib0005] Alghamdi M.A., Elbaz M.I., Ismail I.E., Reda F.M., Alagawany M., El-Tarabily K.A., Abdelgeliel A.S. (2024). Dietary supplementation with a mixture of Dunaliella salina and Spirulina enhances broiler performance by improving growth, immunity, digestive enzymes and gut microbiota. Poult. Sci..

[bib0006] Altmann B.A.C.N., Velten S., Liebert F., Mörlein D. (2018). Meat quality derived from high inclusion of a micro-alga or insect meal as an alternative protein source in poultry diets: a pilot study. Foods.

[bib0007] Alwaleed E.A., El-Sheekh M., Abdel-Daim M.M., Saber H. (2021). Effects of Spirulina platensis and Amphora coffeaeformis as dietary supplements on blood biochemical parameters, intestinal microbial population, and productive performance in broiler chickens. Environ. Sci. Pollut. Res. Int..

[bib0008] Austic R.E., Mustafa A., Jung B., Gatrell S., Lei X.G. (2013). Potential and limitation of a new defatted diatom microalgal biomass in replacing soybean meal and corn in diets for broiler chickens. J. Agric. Food Chem..

[bib0009] Aviagen (2019). Ross 708 broiler: nutrition specification. https://en.aviagen.com/assets/Tech_Center/Ross_Broiler/RossBroilerNutritionSpecs2019-EN.pdf.

[bib0010] Bafundo K.W., Männer K., Duerr I. (2021). The combination of quillaja and yucca saponins in broilers: effects on performance, nutrient digestibility and ileal morphometrics. Br. Poult. Sci..

[bib0011] Bhowmik D., Dubey J., Mehra S. (2009). Probiotic efficiency of Spirulina platensis: stimulating growth of lactic acid bacteria. Am. Eurasian J. Agric. Environ. Sci..

[bib0012] Callahan B.J., McMurdie P.J., Rosen M.J., Han A.W., Johnson A.J., Holmes S.P. (2016). Dada2: high-resolution sample inference from illumina amplicon data. Nat. Methods.

[bib0013] Camacho F., Macedo A., Malcata F. (2019). Potential industrial applications and commercialization of microalgae in the functional food and feed industries: a short review. Mar. Drugs.

[bib0014] Cavitt L.C., Youn G.W., Meullent J.F., Owens C.M., Xiong R. (2004). Prediction of poultry meat tenderness using razor blade shear, Allo-Kramer shear, and sarcomere length. J. Food Sci..

[bib0015] Chamorro-Cevallos G. (2016). Methods for extraction, isolation and purification of cphycocyanin: 50 years of research in review. Int. J. Food Nut. Sci..

[bib0016] Chee S.H., Iji P.A., Choct M., Mikkelsen L.L., Kocher A. (2010). Characterisation and response of intestinal microflora and mucins to manno-oligosaccharide and antibiotic supplementation in broiler chickens. Br. Poult. Sci..

[bib0017] Coelho D., Lopes P.A., Cardoso V., Ponte P., Brás J., Madeira M.S., Alfaia C.M., Bandarna N.M., Gerken H.G., Fontes C.M.G.A., Prates J.A.M. (2019). Novel combination of feed enzymes to improve the degradation of chlorella vulgaris recalcitrant cell wall. Sci. Rep..

[bib0018] Cohen Z., Vonshak A. (1997). Spirulina Platensis (Arthrospira): physiology, Cell-Biology and Biotechnology.

[bib0019] Connolly G., Campbell W.W. (2023). Poultry consumption and Human cardiometabolic health-related outcomes: a narrative review. Nutrients.

[bib0020] Cooke B.C. (1987). The impact of declaration of the metabolizable energy (ME) value of poultry feeds. Recent Adv. Anim. Nutr..

[bib0021] Costa M.M., Spínola M.P., Tavares B., Pestana J.M., Tavares J.C., Martins C.F., Alfaia C.M., Carvalho D.F.P., Mendes A.R., Ferreira J.I., Mourato M.P., Lordelo M.M., Prates J.A.M. (2024). Effects of high dietary inclusion of Arthrospira platensis, either extruded or supplemented with a super-dosing multi-enzyme mixture, on broiler growth performance and major meat quality parameters. BMC Vet. Res..

[bib0022] Cross H.R., West R.L., Dutson T.R. (1981). Comparison of methods for measuring sarcomere length in beef semitendinosis. Meat Sci..

[bib0023] De la Jara A., Ruano-Rodriguez C., Polifrone M., Assunçao P., Brito-Casillas Y., Wägner M., Serra-Majem L. (2018). Impact of dietary arthrospira (spirulina) biomass consumption on human health: main health targets and systematic review. J. Appl. Phycol..

[bib0024] de Mule´ M.C.Z., de Caire G.Z., de Cano M.S. (1996). Bioactive substances from Spirulina platensis (Cyanobacteria). Phyton.

[bib0025] Deng R., Chow T.-J. (2010). Hypolipidemic, antioxidant and antiinflammatory activities of microalgae spirulina. Cardiovasc. Ther..

[bib0026] Dolganyuk V., Belova D., Babich O., Prosekov A., Ivanova S., Katserov D., Patyukov N., Sukhikh A.S. (2020). Microalgae: a promising source of valuable bioproducts. Biomolecules.

[bib0027] Drews G., Carr N.G., Whitton B.A. (1973). The Biology of Blue-green Algae.

[bib0028] El-Bahr S., Shousha S., Shehab A., Khattab W., Ahmed-Farid O., Sabike I., El-Garhy O., Albokhadiam I., Albosadah K. (2020). Effect of dietary microalgae on growth performance, profiles of amino and fatty acids, antioxidant status, and meat quality of broiler chickens. Animals.

[bib0029] El-Deek A.A., Abdel-Wareth A.A., Osman M., El-Shafey M., Khalifah A.M., Elkomy A.E., Lohakare J. (2020). Alternative feed ingredients in the finisher diets for sustainable broiler production. Sci. Rep..

[bib0030] Evans A.M., Smith D.L., Mortz J.S. (2015). Effects of algae incorporation into broiler starter diet formulations on nutrient digestibility and 3 to 21 d bird performance. J. Appl. Poult. Res..

[bib0031] FAO (2023). https://www.fao.org/poultry-production-products/production/en/.

[bib0032] Feldman A.T., Wolfe D. (2014). Tissue processing and hematoxylin and eosin straining. Methods Mol. Biol..

[bib0033] Fernandes E.A., Martins C.F., Sales J.R., Carvalho D.F.P., Prates J.A.M., Lordelo M.M., Martins L.L., Raymundo A., Almeida A.M. (2020). Impact of a 15% Spirulina (Limnospira platensis) dietary inclusion on productive performance and meat traits in naked neck and fully feathered slow-growing broiler strains. Poult. Sci..

[bib0034] Fletcher D.L. (2002). Poultry meat quality. World’s poult. Sci. J..

[bib0035] Flint H.J., Scott K.P., Louis P., Duncan S.H. (2012). The role of the gut microbiota in nutrition and health. Nat. Rev. Gastroenterol. Hepatol..

[bib0036] Geller L.T., Barzily-Rokni M., Danino T., Jonas O.H., Shental N., Nejman D. (2017). Potential role of intratumor bacteria in mediating tumor resistance to the chemotherapeutic drug gemcitabine. Science.

[bib0037] Han P., Li J., Zhong H., Xie J., Zhang P., Lu Q., Li J., Xu P., Chen P., Leng L., Zhou W. (2021). Anti-oxidation properties and therapeutic potentials of spirulina. Algal Res..

[bib0038] Herath H.M.U.L., Jayawardana B.C., Fernando P.D.S.M., Weththasinghe P. (2023). A meta-analysis of the effects of dietary Spirulina on growth performance of broiler chicken. WPSJ.

[bib0039] Hu J., Li Y., Pakpour S., Wang S., Pan Z., Liu J., Wei Q., She J., Cang H., Zhang R.X. (2019). Dose effects of orally administered spirulina suspension on colonic microbiota in healthy mice. Cell. Infect. Microbiol..

[bib0040] Jeong J.S., Kim I.H. (2014). Effect of Bacillus subtilis C-3102 spores as a probiotic feed supplement on growth performance, noxious gas emission, and intestinal microfora in broilers. Poult. Sci..

[bib0041] Jeong J.Y., Janardhanan K.K., Booren A.M., Harte J.B., Kang I. (2011). Breast meat quality and consumer sensory properties of broiler carcasses chilled by water, air, or evaporative air. Poult. Sci..

[bib0042] Kaushik P., Chauhan A. (2008). In vitro antibacterial activity of laboratory grown culture of spirulina platensis. Ind. J. Microbiol..

[bib0043] Kawamura K., Ma D., Pereira A., Ahn D., Kim D., Kang I. (2023). Subzero saline chilling with or without prechilling in icy water improved chilling efficiency and meat tenderness of broiler carcasses. Poult. Sci..

[bib0044] Khambaulai O., Yamauchik L., Tangaweewipt S., Cheva-Isarakul B. (2009). Growth performance and intestinal histology in broiler chicks and with dietary chitosan. Vet. Res..

[bib0045] Khan S., Mobashar M., Mahsood F.K., Javaid S., Abdel-Wareth A.A., Ammanullah H., Mahmood A. (2020). Spirulina inclusion levels in a broiler ration: evaluation of growth performance, gut integrity, and immunity. Trop. Anim. Health Prod..

[bib0046] Koohmaraie M., Geesink G.H. (2006). Contribution of postmortem muscle biochemistry to the delivery of consistent meat quality with particular focus on the calpain system. Meat Sci..

[bib0047] Lopez-Otín C., Blasco M.A., Partridge L., Serrano M., Kroemer G. (2013). The hallmarks of aging. Cell.

[bib0048] Lum K., Kim J., Lei X. (2013). Dual potential of microalgae as a sustainable biofuel feedstock and animal feed. J. Anim. Sci. Biotechnol..

[bib0049] Madeira M.S., Cardoso C., Lopes P.A., Coelho D., Afonso C., Bandarra N.M., Prates J.A. (2017). Microalgae as feed ingredients for livestock production and meat quality: a review. Livest. Sci..

[bib0050] Mahmoud E., Elsayed G., Hassan A., Ateya A., El-Sayed S.A.E. (2025). Dietary spirulina platensis a promising growth promotor and immune stimulant in broiler chickens. Nat. Prod. Res..

[bib0051] Martins C.F., Ribeiro D.M., Costa M., Coelho D., Alfaia C.M., Lordelo M., Almeida A.M., Freire J.P.B., Prates J.A.M. (2021). Using microalgae as a sustainable feed resource to enhance quality and nutritional value of pork and poultry meat. Foods.

[bib0052] Masuda T., Goldsmith P.D. (2009). World soybean production: area harvested, yield, and long-term projections. Int. Food Agribus Manag. Rev..

[bib0053] Metheny M.M., Lee H.C., Viliani S., Bennett D., Hurley S., Kang I. (2019). Improvement of chilling efficiency and meat tenderness of broiler carcasses using sub-zero saline solutions. Poult. Sci..

[bib0054] Moujahed A.R., Hassani S., Zairai S., Bouallegue M., Darej C., Haddad B., Damergi C. (2011). Effect of dehydrated Spirulina platensis on performances and meat quality of Broilers. ROAVS.

[bib0055] Moustafa E.S., Alsanie W.F., Gaber A., Kamel N.N., Alaqil A.A., Abbas A.O. (2021). Blue-green algae (Spirulina platensis) alleviates the negative impact of heat stress on broiler production performance and redox status. Animals.

[bib0056] Mullenix G.J., Maynard C.J., Owens C.M., Rochell S.J., Bottje W.G., Brister R.D., Kidd M.T. (2022). Spirulina platensis meal inclusion effects on broilers fed a reduced protein diet. J. Appl. Poult. Res..

[bib0057] Nagappan S., Das P., AbdulQuadir M., Thaher M., Khan S., Mahata C., Al-Jabri H., Vatland A.K., Kumar G. (2021). Potential of microalgae as a sustainable feed ingredient for aquaculture. J. Biotechnol..

[bib0058] Nam K.C., Lee E.J., Ahn D.U., Ricke S. (2016). The Colour of Poultry Meat: understanding, Measuring and Maintaining Product Quality. Pages 273–290 in Achieving Sustainable Production of Poultry Meat.

[bib0059] Neumann C., Velten S., Liebert F. (2018). The graded inclusion of algae (Spirulina platensis) or insect (Hermetia illucens) meal as a soybean meal substitute in meat type chicken diets impacts on growth, nutrient deposition and dietary protein quality depending on the extent of amino acid supplementation. Open J. Anim. Sci..

[bib0060] Neyrinck A.M., Taminiau B., Walgrave H., Daube G., Cani P.D., Bindels L.B., Delzenne N.M. (2017). Spirulina protects against hepatic inflammation in aging: an effect related to the modulation of the gut microbiota?. Nutrients.

[bib0061] O’Lear Reid T.K., Gardner K.E., Paglia K.L., Ulans A.M.M., Spierling R.E., Edwards M.S., Lundguist T.J., McFarlane Z.D., Pokharel S., Bennett D.C. (2024). Evaluation of apparent metabolizable energy and apparent ileal amino acid digestibility of spirulina (Arthrospira platensis) in broiler chickens and laying hens. Animals.

[bib0062] Papp R.E., Hasenegger V., Ekmekcioglu C., Schwingshackl L. (2023). Association of poultry consumption with cardiovascular diseases and all-cause mortality: a systematic review and dose response meta-analysis of prospective cohort studies. Crit. Rev. Food Sci. Nutr..

[bib0063] Park J., Lee S., Kim I.S. (2018). Effect of dietary Spirulina (Arthrospira) platensis on the growth performance, antioxidant enzyme activity, nutrient digestibility, cecal microflora, excreta noxious gas emission, and breast meat quality of broiler chickens. Poult. Sci..

[bib0064] Pestana J.M., Puerta B., Santos H., Madeira M.S., Alfaia C.M., Lopes P.A., Pinto R.M.A., Lemos J.P.C., Fontes C.M.G.A., Lordelo M.M., Prates J.A.M. (2020). Impact of dietary incorporation of Spirulina (Arthrospira platensis) and exogenous enzymes on broiler performance, carcass traits, and meat quality. Poult. Sci..

[bib0065] Rasool M., Sabina E.P., Lavanya B. (2006). Anti-inflammatory effect of spirulina fusiformis on adjuvant-induced arthritis in mice. Biol. Pharm. Bull..

[bib0066] Ross E., Dominy W. (1990). The nutritional value of dehydrated, blue-green algae (spirulina plantensis) for poultry. Poult. Sci..

[bib0067] Sams A.R., Janky D.M. (1986). The influence of brine chilling on tenderness of hot-boned, chilled-boned, and age-boned broiler fillets. Poult. Sci..

[bib0068] Sansawat T., Lee H.C., Singh P., Kim H., Chin K.B., Kang I. (2014). Combination of muscle tension and crust-freeze-air-chilling improved efficacy of air chilling and quality of broiler fillets. Poult. Sci..

[bib0069] Savage T.F., Zakrzewska E.I., Lyons T.P., Jacques K.A. (1996). Proceedings of the Alltech’s 12th Annual Symposium.

[bib0070] Sharmin F., Sarker N.R., Sarker M.D.S.K.A. (2020). Effect of using moringa oleifera and spirulina platensis as feed additives on performance, meat composition and oxidative stability and fatty acid profiles in broiler chicken. J. Nutr. Food Sci..

[bib0071] Shanmugapriya B., Babu S.S., Hariharan T., Sivaneswaran S., Anusha M.B., Raja P.U. (2015). Synergistic effect of spirulina platensis on performance and gut microbial load of broiler chicks. Indo-Asian J. Multidiscip. Res..

[bib0072] Spínola M.P., Costa M.M., Tavares B., Pestana J.M., Tavares J.C., Martins C.F., Alfaia C.M., Maciel V., Carvalho D.F.P., Mourato M.P., Lordelo M.M., Prates J.A.M. (2024). Impact of long-term feeding a high level of spirulina combined with enzymes on growth performance, carcass traits and meat quality in broiler chickens. Front. Vet. Sci..

[bib0073] Sugiharto S., Yudiarti T., Isroli I., Widiastuti E. (2018). Effect of feeding duration of Spirulina platensis on growth performance, haematological parameters, intestinal microbial population and carcass traits of broiler chicks. S. Afr. J. Anim. Sci..

[bib0074] Sujiwo J.D.K., Jang A. (2018). Relation among quality traits of chicken breast meat during cold storage: correlations between freshness traits and torrymeter values. Poult. Sci..

[bib0075] Teng P.Y., Adhikari R., Llamas-Moya S., Kim W.K. (2021). Effects of combination of mannan-oligosaccharides and b-glucan on growth performance, intestinal morphology, and immune gene expression in broiler chickens. Poult. Sci..

[bib0076] Teng P.Y., Chang C.L., Huang C.M., Chang S.C., Lee T.T. (2017). Effects of solid-state fermented wheat bran by Bacillus amyloliquefaciens and saccharomyces cerevisiae on growth performance and intestinal microbiota in broiler chickens. Ital. J. Anim. Sci..

[bib0077] Tezil T., Basaga H. (2014). Modulation of cell death in age-related diseases. Curr. Pharm. Des..

[bib0078] Toyomizu M.K.S., Taroda H., Kato T., Akiba Y. (2001). Effects of dietary spirulina on meat colour in muscle of broiler chickens. Br. Poult. Sci..

[bib0079] United Nations (2019). 9.7 billion on Earth by 2050, but growth rate slowing, says new UN population report | UN News. https://news.un.org/en/story/2019/06/1040621.

[bib0080] USDA-Food Safety and Inspection Service (2001).

[bib0081] Venkateswarlu S., Devasena B., Sudheer K. (2023). Effect of dietary incorporation of spirulina platensis on the growth performance, carcass characteristics, and meat quality in broilers. J. Meat Sci..

[bib0082] Venkataraman L.V., Somasekaran T., Becker E.W. (1994). Replacement value of blue- green alga (spirulina platensis) for fishmeal and a vitamin-mineral premix for broiler chicks. Br. Poult. Sci..

[bib0083] Wang J., Wu Y., Zhou T., Feng Y., Li L. (2025). Common factors and nutrients affecting intestinal villus height -A review. Anim. Biosci..

[bib0084] Yusuf M.S., Hassan M.A., Abdel-Daim M.M., El Nabtiti A.S., Ahmed A.M., Moawed S.A., El-Sayed K., Cui H. (2016). Value added by Spirulina platensis in two different diets on growth performance, gut microbiota, and meat quality of Japanese quails. Vet. World.

